# Perceptual Restoration of Temporally Distorted Speech in L1 vs. L2: Local Time Reversal and Modulation Filtering

**DOI:** 10.3389/fpsyg.2018.01749

**Published:** 2018-09-19

**Authors:** Mako Ishida, Takayuki Arai, Makio Kashino

**Affiliations:** ^1^NTT Communication Science Laboratories, Atsugi, Japan; ^2^Japan Society for the Promotion of Science, Tokyo, Japan; ^3^Department of Information and Communication Sciences, Sophia University, Tokyo, Japan

**Keywords:** speech perception, perceptual restoration, temporal processing, locally time-reversed speech, modulation filtering, L1, L2

## Abstract

Speech is intelligible even when the temporal envelope of speech is distorted. The current study investigates how native and non-native speakers perceptually restore temporally distorted speech. Participants were native English speakers (NS), and native Japanese speakers who spoke English as a second language (NNS). In Experiment 1, participants listened to “locally time-reversed speech” where every x-ms of speech signal was reversed on the temporal axis. Here, the local time reversal shifted the constituents of the speech signal forward or backward from the original position, and the amplitude envelope of speech was altered as a function of reversed segment length. In Experiment 2, participants listened to “modulation-filtered speech” where the modulation frequency components of speech were low-pass filtered at a particular cut-off frequency. Here, the temporal envelope of speech was altered as a function of cut-off frequency. The results suggest that speech becomes gradually unintelligible as the length of reversed segments increases (Experiment 1), and as a lower cut-off frequency is imposed (Experiment 2). Both experiments exhibit the equivalent level of speech intelligibility across six levels of degradation for native and non-native speakers respectively, which poses a question whether the regular occurrence of local time reversal can be discussed in the modulation frequency domain, by simply converting the length of reversed segments (ms) into frequency (Hz).

## Introduction

People are capable of perceptually restoring temporally distorted speech. Earlier studies suggest that people can perceptually restore a part of speech that is physically missing from the speech signal (Cherry, 1953; Broadbent, 1954; Cherry and Wiley, 1967; Warren, 1970; Warren and Warren, 1970; Warren and Obusek, 1971). The studies of phonemic restoration, where the target phoneme was deleted and filled by the sound of coughs or noise, suggest that people are usually unaware of the replaced part of speech. Rather, they perceptually fill the gap of speech unconsciously, and understand speech with no difficulties. A series of studies suggest that people seem to perceptually restore the deleted phoneme by relying on the acoustic cues, lexicality, and sentential contexts (Warren and Obusek, 1971; Houtgast, 1972; Warren et al., 1972; Warren and Sherman, 1974; Ganong, 1980; Samuel, 1981a,b, 1996; Bashford et al., 1992; Kashino, 1992, 2006; Kashino et al., 1992). As for the acoustic cues, phonemic restoration takes place under certain conditions – when the replacing sound is louder than the original sound (Warren and Warren, 1970; Warren et al., 1972), when the center frequency of the replacing and replaced sound is matched (Warren and Warren, 1970; Warren et al., 1972), and when the replacing sound has similar temporal, spectral, and spatial characteristics as the original sound (Samuel, 1981a,b, 1996; Kashino, 2006). Both native and non-native speakers perceptually restore the missing phoneme when acoustic conditions are met. As for lexicality, the missing phoneme is generally better restored in words than pseudowords by native speakers but similarly restored by non-native speakers (Ishida and Arai, 2015, 2016). The lexical advantage seems to reflect the initial gap of vocabulary size (Hirsch and Nation, 1992; Tinkham, 1993; Nation, 2001, 2006), giving native speakers an advantage in filling the gap in speech (Kashino et al., 1992; Kashino and Craig, 1994; Samuel and Frost, 2015). As for sentential contexts (which were studied mainly with native speakers), the missing phoneme is better restored when presented in semantically coherent sentences than semantically incoherent sentences (Warren and Obusek, 1971; Warren and Sherman, 1974). Previous studies suggest that acoustic characteristics, lexical context, and linguistic coherence are all taken into account consciously or unconsciously, when perceptually restoring the deleted part of speech.

The perceptual restoration of distorted speech also takes place at a larger scale. Saberi and Perrott (1999) suggested that people could understand “locally time-reversed speech” where every x-ms of the speech signal was reversed on the temporal axis. They reported that people could understand speech almost perfectly when every 50 ms was flipped in time, and the rated intelligibility dropped only by half when every 130 ms was locally time-reversed. Speech only became unintelligible when every 200 ms was inverted. People seem to be able to perceptually retrieve acoustic information that was shifted from the original position by the local time reversal, and piece the parts together to perceptually restore the original speech. In other words, the acoustic components of speech can be dispersed from their original positions on the temporal axis, as long as they stay in the restorable range. One thing that should be noted here is that the above mentioned experiment measured the intelligibility of speech by asking for subjective judgments – i.e., people read the target speech before listening to it, and judged the intelligibility of locally time-reversed speech subjectively. The repetitive exposure to the target speech might have produced the relatively high intelligibility ratings for relatively long temporal reversal (Stilp et al., 2010; Remez et al., 2013; Ueda et al., 2017), but this study showed, at least, that people might be tolerant to temporal distortion occurring at a large scale. Their findings raised the question of whether detailed analysis of the temporal fine structure of speech is required in speech perception (Liberman et al., 1967; Steffen and Werani, 1994; Greenberg, 1999; Greenberg and Arai, 2001; Magrin-Chagnolleau et al., 2002; Remez et al., 2013; Ueda et al., 2017). Rather, speech perception might take place at a slower pace in the range of 3–8 Hz in the modulation frequency domain, or 125–333 ms (Remez et al., 2013), which is comparable to the pace of syllable production (Huggins, 1964, 1975; Arai and Greenberg, 1997; Greenberg et al., 1998; Greenberg, 1999; Grataloup et al., 2009; Stilp et al., 2010).

Greenberg and Arai (2001) further investigated the relationship between the intelligibility of speech and local time reversal by adopting an objective measurement – dictation. In general, locally time-reversed speech became gradually unintelligible as the length of reversed segments increased, as was also reported in Saberi and Perrott (1999). However, the intelligibility of speech dropped by half already when the reversed segment length was 60 ms, and speech became unintelligible when the reversed segment length was 100 ms. Subsequent studies of locally time-reversed speech also supported these results, with objective measurement, that speech became unintelligible when the reversed segment length was shorter than 100 ms. These results were shown in English (Stilp et al., 2010; Remez et al., 2013; Ishida et al., 2016; Ishida, 2017; Ueda et al., 2017), French (Magrin-Chagnolleau et al., 2002), German (Kiss et al., 2008; Ueda et al., 2017), Japanese (Ueda et al., 2017), and Mandarin Chinese (Ueda et al., 2017). These results added to the discussion of whether the detailed analysis of temporal fine structure is required or not in speech perception. The perceptual organization of speech might take place at a relatively fast pace, too, which is comparable to the pace of phoneme production.

In fact, many studies of locally reversed speech focus on speech intelligibility in relation to the reversed segment length (Saberi and Perrott, 1999; Greenberg and Arai, 2001; Magrin-Chagnolleau et al., 2002; Kiss et al., 2008; Stilp et al., 2010; Remez et al., 2013; Ueda et al., 2017). The reversed segment length, which was measured in duration (ms), is further converted into frequency (Hz), in order to discuss which modulation frequency is critical for speech intelligibility. The regular occurrence of temporal reversal (e.g., 125–333 ms) is regarded as a property that modulates the temporal envelope of speech, and discussed in the modulation frequency domain (e.g., 3–8 Hz) (Saberi and Perrott, 1999; Remez et al., 2013). Is this appropriate? The reversed segment length is also regarded as a reflection of the critical temporal range or boundary of how much acoustic elements of speech signal can be dispersed from their original temporal position by the local time reversal, so that they can be integrated for perceptual restoration. Put another way, the reversed segment length is discussed as a basic unit of information to be integrated in the perceptual restoration of speech. Further, the basic unit of information, measured in milliseconds, has been discussed in relation to linguistic properties – as in whether speech perception takes place at a pace of a syllable or a phoneme or both, or if speech is perceived based on the accumulation of relatively short or long temporal information that is comparable to syllable or phoneme durations. Is this appropriate? Greenberg and Arai (2001) analyzed the envelope-modulation patterns of locally time-reversed speech as compared to the original speech – i.e., how much the amplitude and phase components of the speech signal were altered or dispersed as a result of local time reversal. They computed the modulation magnitude by dissociating the amplitude and phase components first, and later integrating them to form the “complex modulation spectrum.” Their analysis showed that the modulation frequency between 3 and 8 Hz is critical for speech intelligibility, although the intelligibility of locally time-reversed speech dropped by half when the reversed segment length was 60 ms, and speech became unintelligible when the reversed segment length was greater than 100 ms. In fact, the reversed segment length of 60 – 100 ms corresponds to 10 – 17 Hz, and this does not match the critical modulation frequency range of 3 – 8 Hz. This seems to suggest that the direct conversion from the duration of temporal reversal (ms) into frequency (Hz) might not be an appropriate way to talk about the critical modulation frequency for the intelligibility of locally time-reversed speech. This needs to be further investigated.

Moreover, language proficiency also affects the perceptual restoration of temporally distorted speech. Kiss et al. (2008) suggested that both native and non-native speakers perceptually restored semantically coherent sentences better (where sentential and lexical contexts were available) than semantically incoherent sentences (where only lexical context was available) when locally time-reversed. However, a significant difference was only observed with native speakers and not with non-native speakers. It seems that native speakers can benefit from both sentential and lexical context greatly when perceptually restoring degraded speech (Miller and Isard, 1963; Treisman, 1969; Davis et al., 2005), while non-native speakers primarily relied on lexical context for perceptual restoration. On the other hand, both native and non-native speakers barely understood pseudo-homophonic sentences (where no semantic context was available but a phonotactically legal sequence of sounds was available) when locally time-reversed. The basic acoustic information seems to be similarly processed by both native and non-native speakers. It seems to suggest that lexical knowledge influences perceptual restoration greatly, especially when the temporal distortion occurs in the listener’s second language (Warren and Warren, 1970; Saragi et al., 1978; Nation, 2001; Ishida and Arai, 2015, 2016; Ishida, 2017). In fact, native speakers of English are presumed to have a reading vocabulary of 40,000–80,000 words after graduating from high school (Aitchison, 2012). In contrast, non-native speakers need an 8,000–9,000 word-family vocabulary to understand 98% of written text without any assistance, and a 6,000–7,000 word-family vocabulary to understand 98% of normally spoken material without any assistance (Nation, 2006). The relationship between the vocabulary size of listeners and perceptual restoration of temporally distorted speech should be studied further.

The current study examines how native and non-native speakers of English perceptually restore temporally distorted speech. This study adopts two kinds of temporal distortion – i.e., locally time-reversed speech, and modulation-filtered speech. In Experiment 1, native speakers and non-native speakers listen to locally time-reversed speech where every local segment of speech is flipped in time (i.e., every 10, 30, 50, 70, 90, or 110 ms). Here, the temporal envelope of speech is distorted as a function of reversed segment length. In Experiment 2, native and non-native speakers listen to modulation-filtered speech in which the modulation frequency components are low-pass filtered at a particular cut-off frequency (i.e., 32, 16, 8, 4, 2, or 1 Hz). Here, the temporal envelope of speech is distorted as a function of low-pass cut-off frequency (cf. Drullman et al., 1994), and the temporal fine structure of speech signal is expected to be smeared when lower cut-off frequency is imposed (i.e., when only fewer modulation frequency components are preserved). The modulation filtering is adopted as a means to explore the intelligibility of speech to examine the relationship between the reversed segment length (ms) and modulation frequency (Hz) in parallel. The low-pass filtering is manipulated based on Greenberg et al. (1998), who followed the basic procedure of Drullman et al. (1994). In this approach, the wide band signal of speech is divided into sub-bands, and the amplitude envelope of every sub-band is computed and low-pass filtered at a particular cut-off frequency, and finally put back together to form the amplitude-modulated speech signal (see the details in Experiment 2). The modulation filtering is performed in order to examine which modulation frequency components must be preserved in order to maintain the intelligibility of speech. The same individuals participate in two consecutive experiments. For counterbalancing purposes, half of the participants started with Experiment 1 (locally time-reversed speech) followed by Experiment 2 (modulation-filtered speech), and the other half started with Experiment 2 followed by Experiment 1. The intelligibility of speech is measured as a function of the increase in reversed segment length (Experiment 1), and as a function of the shift in low-pass cut-off frequency (Experiment 2). The perceptual tolerance to the temporal degradation of speech is examined by recruiting native English speakers in the United States (L1 English speakers), and native Japanese speakers in Japan who speak English as a second language (L2 English speakers). These two groups of participants were presumed to have a large difference in vocabulary size (Nation, 2001, 2006), and the English proficiency of non-native speakers was defined based on their approximate vocabulary size.

## Experiment 1

**FIGURE 1:**
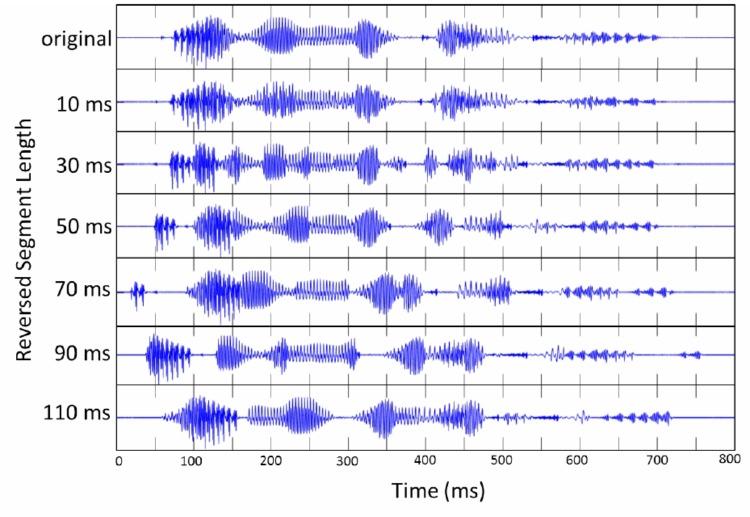
The waveform of “I will look into it” (original) is shown at the top. The waveform of locally time-reversed speech is displayed below next to the corresponding reversed segment length: 10, 30, 50, 70, 90, and 110 ms.

Experiment 1 explores how native and non-native speakers perceptually restore locally time-reversed speech in which every fixed duration of speech signal is flipped in time (**Figure 1**). This experiment examines how native and non-native speakers cope with the regular occurrence of temporal reversal, which entails the alteration of the temporal envelope of speech. The intelligibility of locally time-reversed speech is examined by adopting 6 reversed segment lengths. The reversed segment lengths are selected to induce a gradual change in the temporal envelope of speech. This study examines the intelligibility of locally time-reversed speech as a function of reversed segment length, and the involvement of language proficiency (i.e., vocabulary size) in perceptual restoration of temporal distortion.

### Participants

#### Native Speakers

Thirty native English speakers from Stony Brook University (25 female, 5 male, ave. 19.9 years old) participated in this study. None of them reported any hearing problems. Participants received course credit for their participation. They completed a consent form when they agreed to participate in this study. The consent form was approved by the Institutional Review Board (IRB) of Stony Brook University.

#### Non-native Speakers

Thirty native Japanese speakers who spoke English as a second language (20 female, 10 male, ave. 34.9 years old) participated in this study. All of them started learning English when they entered the junior high school in Japan (12 years old). Their English proficiency was lower intermediate on the DIALANG placement test (Alderson, 2006; Lancaster University, 2014). Their average score was 386 out of 1,000 full marks, and their proficiency level was fourth from the top, out of 6 levels. None reported any hearing or speech impairment. Participants received monetary remuneration for their participation. They completed a consent form when they agreed to participate in this study. The consent form was approved by the Institutional Review Board (IRB) of the NTT Communication Science Laboratories.

### Materials

Eighteen sentences were selected from “Listen, Read, and Write: Sentences for Sight Word Dictation” by Wickham (2013) (**Appendix A**). This book contains 115 sentences for dictation classified from Level 1 to 5, originally designed to train the literacy skills of elementary school students as well as of second language learners. The selected sentences were from Level 1 and 2 (very basic), and contained only high frequency sight words (pronouns, adjectives, prepositions, conjunctions, and verbs) originating from the list of the 220 most frequent words (Dolch, 1948). These words were considered to make up 50–75% of words in books, magazines, and newspapers. Each test sentence contained 3 – 7 words (*M* = 5.3 words). The selected sentences were spoken by a male native speaker of American English, and recorded in a sound proof room by using a microphone (SONY ECM-MS957) and a digital audio recorder (SONY PCM-D50). All stimuli were recorded at a sampling rate of 44.1 kHz with 16-bit resolution. The recorded sounds were then downsampled to 16 kHz (16 bit), and saved as WAV files. The sound level was normalized based on the peak level by using GoldWave digital audio editing software. The speech signals of selected sentences were split into small segments with fixed duration from the onset of speech, and every local segment was reversed on the temporal axis. The reversed segment lengths were 10, 30, 50, 70, 90, or 110 ms (**Figure 1**), which were decided based on previous studies (Greenberg and Arai, 2001; Kiss et al., 2008; Remez et al., 2013; Ishida et al., 2016; Ishida, 2017). The adjacent edges of reversed segments were cross-faded with a tapering length of 5 ms to prevent any additional noise or clicks, as was also adopted in previous studies (Ishida et al., 2016; Ishida, 2017). The local time reversal of speech signal was performed by using MATLAB.

### Procedure

For native speakers, the experiment took place in a sound proof room of Stony Brook University in the United States. Participants listened to the stimuli facing a computer monitor. The stimuli were presented diotically over headphones (SONY MRD-V900HD) at a participant’s comfortable listening level. There was a headphone amplifier (RANE HC6) between the computer’s audio output and the headphones.

For non-native speakers, the experiment took place in a sound proof room of NTT Communication Science Laboratories in Japan. The stimuli were presented diotically over headphones (SONY MDR-CD900ST) at a participant’s comfortable listening level. There was a headphone amplifier (Roland UA-25 EX) between the computer’s audio output and the headphones.

Listeners were instructed to listen to a set of 18 locally time-reversed sentences and transcribe what they heard using a pen and paper. They transcribed each sentence next to the trial number on a separate sheet. Each sentence was followed by 20 s of silence, and participants were asked to transcribe what they heard as soon as they could. Listeners were forced to move on to the next sentence when the presentation of the target sentence and the following 20 s of silence had passed. This procedure was used to prevent listeners from listening to the target sentence multiple times on the spot, and guessing unclear parts by referring to the repetitively presented auditory input or their own transcription. The intention here was to examine perceptual restoration under conditions in which, as in daily situations, listeners have no chance of listening to the whole utterance (a single sentence) multiple times on the spot. After listening to the whole set of sentences (i.e., 18 sentences), participants were asked to listen to the same set of sentences again to check their answers and legibility. Here, listeners were allowed to correct their answers if they wanted to, with a different color pen. The color of the pen was changed from the first listening to the second listening, in order to obtain sentences that were legible (with no entangled letters of the same color).

Each participant listened to the set of 18 sentences, comprised of 6 subsets of 3 sentences, with each subset assigned to one of the 6 reversed segment lengths (10, 30, 50, 70, 90, and 110 ms). Six groups of participants, in a Latin square, were used to counterbalance 6 subsets of items across 6 reversed segment lengths. Before the main experiment, there was a practice session in which participants listened to two locally time-reversed sentences, and practiced transcribing what they heard as soon as they could on a separate sheet next to the trial number. They transcribed what they heard with a blue pen for the first listening, and with a pink pen for the second listening. The total duration of experiment, including the practice session, was approximately 30 min.

### Results and Discussion

**FIGURE 2:**
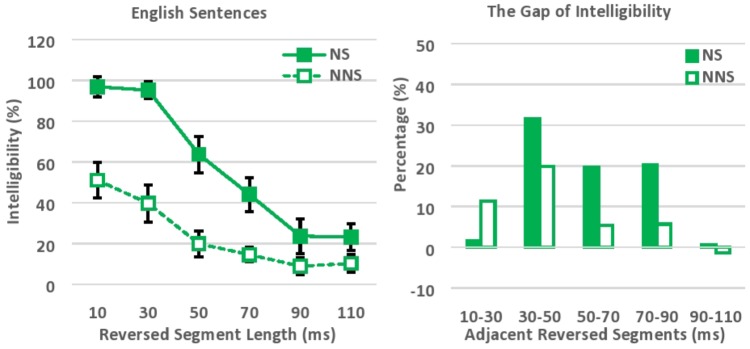
Intelligibility of locally time-reversed English sentences **(left)**, and the gap of intelligibility between adjacent reversed segment lengths **(right)**. NS, Native speakers of English; NNS, Non-native speakers of English (native Japanese speakers who speak English as a second language).

The intelligibility of locally time-reversed sentences was evaluated based on the number of words correctly transcribed (**Figure 2**). Accuracy was 97, 95, 64, 44, 24, and 23% correct for native speakers versus 51, 40, 20, 15, 9, and 10% correct for non-native speakers, when every 10, 30, 50, 70, 90, or 110 ms of speech was flipped in time. In general, native speakers were able to understand locally time-reversed sentences almost perfectly when the reversed segment length was short (10 ms, 30 ms), while non-native speakers understood half of the words in the same speech. This gap seems to reflect the difference in language proficiency between native and non-native speakers (i.e., vocabulary size), although the correlation between non-native listeners’ proficiency level (which was measured in the DIALANG Test where the score is based on the listener’s approximate vocabulary size) and their average performance across all 6 levels of reversed segment length was not significant, *r* = 0.33, *n* = 30, *p* = 0.07. On the other hand, the intelligibility of locally time-reversed sentences drastically dropped for both native and non-native speakers, when the reversed segment length was extended from 30 ms to 50 ms (from 95 to 64% for native speakers, and from 40 to 20% for non-native speakers). Further, the intelligibility dropped to under 50% even for native speakers when the reversed segment length exceeded 70 ms. This seems to follow the general trend that previous studies of locally time-reversed sentences suggested with objective measurement – i.e., the intelligibility of speech gradually declines when the reversed segment length increases, and speech becomes unintelligible even when the reversed segment length is less than 100 ms.

An ANOVA was performed with Greenhouse–Geisser Corrections, with language (native vs. non-native proficiency) as a between-subject factor, and reversed segment length (10, 30, 50, 70, 90, and 110 ms) as a within-subject factor. Native speakers perceptually restored locally time-reversed sentences significantly more than non-native speakers, *F*(1,58) = 201.42, *p* < 0.001, $\eta_{p}^{2}$ = 0.78. In addition, the intelligibility of locally time-reversed sentences dropped significantly when the reversed segment length was extended, *F*(3.68,213.50) = 114.11, *p* < 0.001, $\eta_{p}^{2}$ = 0.66. Here, the *post hoc* pairwise comparisons with Bonferroni corrections suggested that the intelligibility of locally time-reversed speech dropped significantly when the reversed segment length was extended from 30 to 50 ms (*p* < 0.001), from 50 to 70 ms (*p* < 0.05), and from 70 to 90 ms (*p* = 0.001), while the significant intelligibility drop was not observed when the reversed segment length was extended from 10 to 30 ms, and from 90 to 110 ms. There was also a significant interaction between the reversed segment length and language, *F*(3.68,213.50) = 14.00, *p* < 0.001, $\eta_{p}^{2}$ = 0.19. The perceptual restoration by native speakers was much better than that by non-native speakers, especially, when the reversed segment length was short, and, therefore, native speakers were much more affected by the change in segment size than non-native speakers. The follow-up independent *t*-test also suggested that the performance of native and non-native speakers was significantly different across 6 levels of reversed segment length, while the effect size (Cohen’s *d*), along with confidence interval and *t*-value, progressively decreased as the reversed segment length increased (see **Supplementary Table 1**): i.e., *t*(45) = 8.91, *p* < 0.001, 95% CI [0.35 0.56], *d* = 2.27 for 10 ms, *t*(41) = 10.93, *p* < 0.001, 95% CI [0.45 0.66], *d* = 2.80 for 30 ms, *t*(52) = 7.87, *p* < 0.001, 95% CI [0.33 0.55], *d* = 2.02 for 50 ms, *t*(40) = 6.40, *p* < 0.001, 95% CI [0.20 0.39], *d* = 1.64 for 70 ms, *t*(42) = 3.08, *p* = 0.004, 95% CI [0.05 0.24], *d* = 0.80 for 90 ms, and *t*(50) = 3.22, *p* = 0.002, 95% CI [0.05 0.21], *d* = 0.85 for 110 ms. The language proficiency of listeners (i.e., vocabulary size in the current study design) seems to significantly affect the perceptual restoration of locally time-reversed sentences, and the gap of performance between native and non-native speakers progressively decreased as the level of acoustic degradation increased with longer reversed segment length.

Comparing the intelligibility of locally time-reversed sentences here to that of locally time-reversed words (Ishida et al., 2016), lexical items were more intelligible than sentences when the same length of local time reversal was imposed. Presumably, the temporal distortion in short utterances (words) was easier to overcome than the temporal distortion in long utterances (sentences) because of the smaller memory demands (Pisoni, 1973; Mattys et al., 2014). The locally time reversed sentences should have required listeners to retain what they heard (i.e., fragments of speech sounds) until they could reorganize the perceived sounds to make sense of the speech as a whole. The memory demands in the current study design were high, because listeners were able to listen to the target sentence only once on the spot. As for non-native speakers who had a smaller vocabulary size and limited knowledge on semantic collocations, it was possibly difficult to retain what they heard (i.e., multiple words) in their memory on the spot, and think of sentential coherence. Therefore, there were much greater memory demands for the sentence task than the word task, and the greater memory demands with sentences may have had a greater impact on participants operating in L2 than in L1.

## Experiment 2

**FIGURE 3:**
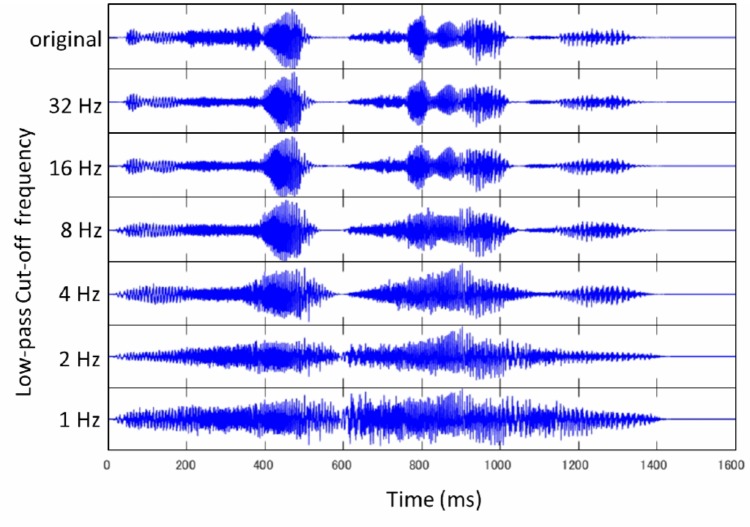
The waveform of “It is so pretty up here” (original) is shown at the top. The waveform of modulation-filtered speech is displayed below next to the corresponding low-pass cut-off frequency: 32, 16, 8, 4, 2, and 1 Hz.

Experiment 2 explores how native and non-native speakers perceptually restore modulation-filtered speech where modulation frequency components are low-pass filtered at a particular cut-off frequency (**Figure 3**). This experiment examines how native and non-native speakers cope with modulation filtering, and the subsequent changes in the temporal envelope of speech. The temporal envelope of speech changes as a function of low-pass cut-off frequency (i.e., preserved modulation frequency components). The temporal fine structure is expected to be gradually lost when lower cut-off frequency is imposed (i.e., when only fewer modulation frequency components are preserved). The intelligibility of speech was examined by adopting 6 low-pass cut-off frequencies, a range selected to induce a gradual change in the temporal envelope of speech (Drullman et al., 1994; Greenberg and Arai, 2001). The perceptual tolerance to the temporal envelope smearing was examined in relation to the language proficiency of listeners (i.e., vocabulary size in the current study design).

### Participants

Participants were the same as in Experiment 1. There were thirty native speakers of English and thirty non-native speakers of English.

### Materials

Eighteen sentences were newly selected from Level 1 and 2 (very basic) of “Listen, Read and Write Sentences for Sight Word Dictation” (Wickham, 2013) (**Appendix B**). These sentences were different from those used in Experiment 1. Each sentence contained 3–8 words (*M* = 5.4 words). These sentences were spoken by the same male speaker as in Experiment 1, and recorded in the same sound proof room using the same apparatus as in Experiment 1. The recorded speech was digitized at 44.1 kHz (16 bit), downsampled to 16 kHz (16 bit), and saved as a WAV file. The temporal envelope of the speech signal was modified based on the procedure used by Greenberg et al. (1998). The original wideband signal (the maximum frequency was set as 6,000 Hz) was first split into 14 frequency bands: 13 1/3-octave-wide channels and the remaining 1 channel using an FIR filter (0–298, 298–375, 375–473, 473–595, 595–750, 750–945, 945–1191, 1191–1500, 1500–1890, 1890–2381, 2381–3000, 3000–3780, 3780–4762, and 4762–6000 Hz). The FIR filter was designed using the Kaiser window (Kaiser, 1966; Oppenheim et al., 1999), in which the transition length was 100 Hz, and the peak approximation error was *δ* = 0.001. The slopes of the FIR filter exceeded 100 dB/oct. The amplitude envelope of each band signal was then computed using the Hilbert Transform (Rabiner and Gold, 1975). The computed envelope was low-pass filtered using another FIR filter designed with the Kaiser window. The transition length was 1 Hz, the peak approximation error was *δ* = 0.1, and the low-pass cut-off frequency was 32, 16, 8, 4, 2, or 1 Hz. The modified band signals were put back together to form the amplitude-modulated speech signals (**Figure 3**). Here, the range of 6 low-pass cut-off frequencies was chosen based on Drullman et al. (1994) and Greenberg and Arai (2001) which suggested the importance of lower modulation frequencies for speech intelligibility. The sound level was normalized based on the RMS value. All the manipulations were performed by MATLAB.

### Procedure

The current study followed a procedure similar to Experiment 1. All of the experiment devices and conditions (for both native and non-native speakers) were the same as those described in Experiment 1.

Listeners were instructed to listen to a set of 18 sentences in which the modulation frequency components were low-pass filtered at a particular cut-off frequency. They transcribed what they heard next to the trial number on a separate sheet. All sentences were presented with 20-s inter-stimulus-intervals. Listeners were instructed to move on to the next sentence after the presentation of the target sentence and the following 20 s had passed. After listening to the set of sentences, listeners were asked to listen to the same set of sentences again to check their answers and legibility. They were allowed to correct their answers if they wanted to in the second listening, with a different color pen. Each subject listened to a set of 18 sentences, comprised of 6 subsets of 3 sentences, with each subset assigned to one of the 6 low-pass cut-off frequencies (32, 16, 8, 4, 2, or 1 Hz). Six groups of participants, in a Latin square, were used to counterbalance 6 subsets of items across 6 cut-off frequencies. Before the main experiment, there was a practice session. Participants listened to a set of 2 modulation-filtered sentences, and practiced transcribing what they heard as soon as they could on a separate sheet. They transcribed what they heard with a blue pen for the first listening, and with a pink pen for the second listening. The total duration of experiment, including the practice session, was approximately 30 min.

### Results and Discussion

**FIGURE 4:**
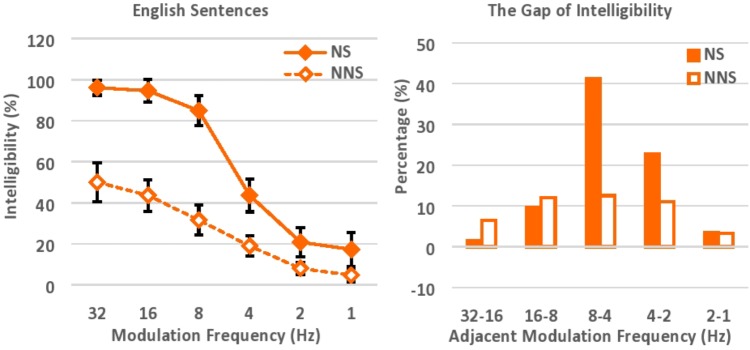
Intelligibility of English sentences with various low-pass cut-off frequencies **(left)**, and the gap of intelligibility between adjacent cut-off frequencies **(right)**. NS, Native speakers of English; NNS, Non-native speakers of English (native Japanese speakers who speak English as a second language).

The intelligibility of speech was evaluated based on the number of words correctly transcribed (**Figure 4**). Accuracy was 96, 95, 85, 44, 21, and 17% correct for native speakers, versus 50, 44, 32, 19, 8, and 5% correct for non-native speakers, when the modulation frequency components of speech were low-pass filtered at the cut-off frequencies of 32, 16, 8, 4, 2, and 1 Hz respectively. In general, native speakers were able to understand modulation-filtered speech almost perfectly when the modulation frequency components were relatively preserved with higher cut-off frequencies (e.g., 32 Hz, 16 Hz), while non-native speakers understood half of the words in the same speech. As was also seen in Experiment 1, this gap between native and non-native speakers reflects the different linguistic competence, particularly in vocabulary size in the current study design, for perceptual restoration (Miller and Isard, 1963; Warren and Warren, 1970; Saragi et al., 1978; Nation, 2001; Ishida and Arai, 2015, 2016; Ishida, 2017). The correlation between non-native listeners’ proficiency level (which was measured in the DIALANG Test where the score is based on the listener’s approximate vocabulary size) and their average performance across all 6 levels of low-pass cut-off frequency was also significant (*r* = 0.40, *n* = 30, *p* < 0.05). The intelligibility of modulation-filtered speech dropped precipitously for both native and non-native speakers, when the cut-off frequency was shifted from 8 Hz to 4 Hz (from 85 to 44% for native speakers, and from 32 to 19% for non-native speakers). The intelligibility dropped to under 50% for native speakers when the cut-off frequency was lower than 4 Hz. As in Experiment 1, the intelligibility of speech gradually declined as higher degradation was imposed with lower cut-off frequencies. The language proficiency of listeners (i.e., native vs. non-native proficiency) affected the perceptual restoration of modulation-filtered speech, and native speakers were able to understand degraded speech better than non-native speakers.

An ANOVA was performed with Greenhouse–Geisser corrections, with language (native vs. non-native speakers) as a between-subject factor, and low-pass cut-off frequency (32, 16, 8, 4, 2, and 1 Hz) as a within-subject factor. Native speakers understood modulation-filtered speech significantly better than non-native speakers, *F*(1,58) = 211.38, *p* < 0.001, $\eta_{p}^{2}$ = 0.79. In addition, speech was significantly more intelligible when higher cut-off frequency was imposed, *F*(2.98,172.89) = 142.12, *p* < 0.001, $\eta_{p}^{2}$= 0.71. Here, the *post hoc* pairwise comparisons with Bonferroni corrections suggested that the intelligibility of speech dropped significantly when the low-pass cut-off frequency was shifted from 16 to 8 Hz (*p* < 0.001), 8 to 4 Hz (*p* < .001), and 4 to 2 Hz (*p* < 0.001), while the significant intelligibility drop was not observed when the low-pass cut-off frequency was shifted from 32 to 16 Hz, and from 2 to 1 Hz. There was also a significant interaction between language and cut-off frequency, *F*(2.98,172.89) = 16.89, *p* < 0.001, $\eta_{p}^{2}$ = 0.23. Overall, native speakers were much more affected by the shift of modulation cut-off frequency when the preserved modulation frequency components declined, especially because their initial performance with higher cut-off frequency was relatively high as compared to that of non-native speakers. The follow-up independent *t*-test also suggested that the performance of native and non-native speakers was significantly different across 6 levels of low-pass cut-off frequency, while the effect size (Cohen’s *d*), along with confidence interval and *t*-value, progressively decreased as lower cut-off frequency was imposed (see **Supplementary Table 2**): i.e., *t*(38) = 8.82, *p* < 0.001, 95% CI [0.36 0.57], *d* = 2.31 for 32 Hz, *t*(53) = 10.50, *p* < 0.001, 95% CI [0.41 0.61], *d* = 2.65 for 16 Hz, *t*(58) = 10.08, *p* < 0.001, 95% CI [0.43 0.64], *d* = 2.58 for 8 Hz, *t*(58) = 5.09, *p* < 0.001, 95% CI [0.15 0.34], *d* = 1.36 for 4 Hz, *t*(38) = 3.31, *p* = 0.002, 95% CI [0.05 0.21], *d* = 0.85 for 2 Hz, and *t*(37) = 2.73, *p* = 0.01, 95% CI [0.03 0.22], *d* = 0.66 for 1 Hz. The language proficiency of listeners seems to significantly affect the perceptual restoration of modulation-filtered speech, and the gap of performance between native and non-native speakers progressively decreased as the level of acoustic degradation increased with lower cut-off frequency.

**FIGURE 5:**
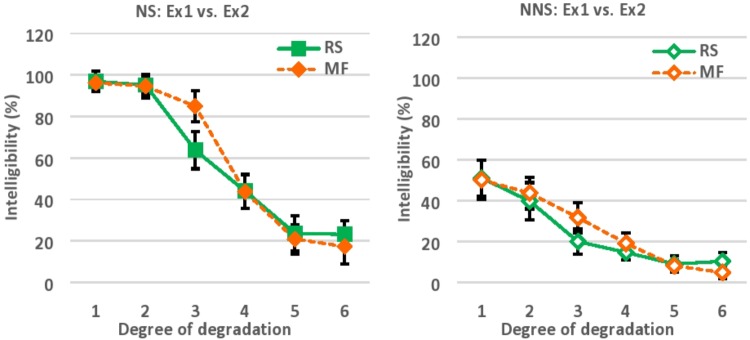
The results of Experiment 1 and 2 for NS is plotted together **(left)**, and the results of Experiment 1 and 2 for NNS is plotted together **(right)** for comparison. NS, Native speakers of English; NNS, Non-native speakers of English (native Japanese speakers who speak English as a second language), Ex1, Experiment 1; Ex2, Experiment 2; RS, Locally time-reversed speech (Experiment 1); MF, Modulation-filtered speech (Experiment 2); Degree of degradation, 1 (10 ms, 32 Hz), 2 (30 ms, 16 Hz), 3 (50 ms, 8 Hz), 4 (70 ms, 4 Hz), 5 (90 ms, 2 Hz), 6 (110 ms, 1 Hz).

Comparing the results of Experiment 1 and 2, the equivalent level of intelligibility was obtained as a function of reversed segment length, and low-pass cut-off frequency across 6 levels of degradation (**Figure 5**). To recall, the intelligibility of locally time-reversed speech (Experiment 1) was 97, 95, 64, 44, 24, and 23% for native speakers vs. 51, 40, 20, 15, 9, and 10% for non-native speakers, when every 10, 30, 50, 70, 90, or 110 ms of speech was flipped in time. Also, the intelligibility of modulation-filtered speech (Experiment 2) was 96, 95, 85, 44, 21, and 17% for native speakers, vs. 50, 44, 32, 19, 8, and 5% for non-native speakers, when the modulation frequency components of speech were low-pass filtered at the cut-off frequencies of 32, 16, 8, 4, 2, and 1 Hz respectively. There was an initial gap between native and non-native speakers when the mildest degradation was imposed with the reversed segment length of 10 ms (97% vs. 51% for NS vs. NNS) in Experiment 1, and with the cut-off frequency of 32 Hz (96 % vs. 50% for NS vs. NNS) in Experiment 2. Presumably, this gap reflects the difference of language proficiency between native and non-native speakers, since the gap amount was almost equivalent in two different tasks. The language proficiency of NNS in the current study was lower intermediate based on the DIALANG placement test which measured the approximate vocabulary size of test takers. There should be a huge gap between native and non-native speakers in terms of vocabulary size as well as listening proficiency. In fact, even without acoustic manipulation of temporal reversal or modulation filtering, second language learners have difficulties in understanding connected speech in daily situations, where a series of adjacent words are combined and pronounced together (Brown and Hilferty, 1982, 1986, 1995; Brown and Kondo-Brown, 2006). Even when a series of words in speech is easy enough to understand (e.g., high-frequency words which were also used in the current study), the accurate perception of speech can be difficult when listeners are not familiar with connected speech. Further, when acoustic distortion is added to connected speech, it should be more difficult for second language learners to understand (Ishida, 2017), which can happen in daily situations as in listening to public announcements in a loud environment, or talking with native speakers over phones.

Lastly, the correlation between non-native listeners’ language proficiency based on the DIALANG Test (i.e., vocabulary size) and their performance in perceptual restoration was not significant in Experiment 1, while the correlation was significant in Experiment 2. It is possible that non-native speakers required a little more vocabulary knowledge for the perceptual restoration of low-pass filtered speech than of locally time-reversed speech, but this remains to be explored further in the future study.

## General Discussion

The current study explored how native and non-native speakers perceptually restore temporally distorted speech by adopting two techniques. Experiment 1 degraded speech by reversing every x-ms of speech signal on the temporal axis (locally time-reversed speech). Here, six reversed segment lengths were adopted for local time reversal: 10, 30, 50, 70, 90, and 110 ms. Speech was intelligible when the reversed segment length was relatively short, and the intelligibility gradually declined as the length of the reversed segment increased. Put another way, speech was intelligible when the regular occurrence of local time reversal did not drastically change the temporal envelope of speech as compared to the original one. However, speech became gradually unintelligible as the regular occurrence of local time reversal altered the temporal envelope of speech. The intelligibility of speech dropped by half when the reversed segment length reached 70 ms in the present study (44% intelligibility), which followed the general trend of previous studies.

Experiment 2 degraded speech by low-pass filtering the modulation frequency components at a particular cut-off frequency (modulation-filtered speech). Here, six cut-off frequencies were adopted for modulation filtering: 32, 16, 8, 4, 2, and 1 Hz. Speech was intelligible when a higher cut-off frequency was imposed, that is, when more modulation frequency components were preserved. The intelligibility gradually declined as lower cut-off frequencies were imposed, and fewer modulation frequency components were preserved. Put another way, speech was intelligible when the low-pass filtering did not change the temporal envelope of speech drastically as compared to the original one. However, speech became gradually unintelligible as the low-pass filtering altered or smeared the fine structure of the temporal envelope of speech. The intelligibility of speech dropped by half when only the modulation frequency components lower than 4 Hz were preserved in the present study (44% intelligibility), which is in the range of 3-8 Hz that previous studies discussed.

The parallel comparison of Experiment 1 and 2 poses a question: Is it appropriate to discuss the duration of local time reversal, measured in milliseconds, in the modulation frequency domain, by directly converting the temporal duration (ms) into frequency (Hz), as was done in some previous studies? While the regular occurrence of local time reversal modulates or alters the temporal envelope of speech, it is likely that the regular temporal inversion also generates additional artifacts that do not exist in natural speech. These additional artifacts can affect the modulation frequency in some way. Even if the edges of reversed segments are smoothed with linear amplitude ramps or other tapering methods, the local time inversion shifts or “disperses” the phase and amplitude components of speech from their original temporal position (Greenberg and Arai, 2001), and connects the acoustic components of speech unnaturally and artificially at the edge. For example, the temporal constituents of speech which are aligned in the order of 0–50 ms and 51–100 ms will be sequenced in the order of 50-0-100-51 when locally time-reversed at every 50 ms (*Note*: this is just a rough explanation of how the reversed segments are unnaturally and artificially connected at the edge). The phase and amplitude components of speech, which are unnaturally and artificially connected, would generate additional noise or clicks, which would affect the modulation frequency of speech.

**FIGURE 6:**
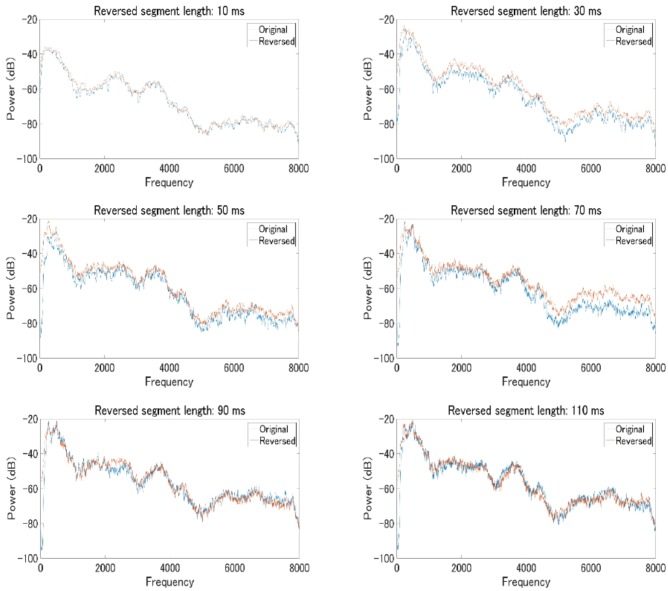
The average power spectrum of 18 original sentences (blue line) and 18 locally time-reversed sentences (red line) across all frequency range, according to the reversed segment length of 10 ms **(top left)**, 30 ms **(top right)**, 50 ms **(middle left)**, 70 ms **(middle right)**, 90 ms **(bottom left)**, and 110 ms **(bottom right)**. The horizontal axis shows frequency range (0–8,000 Hz), and the vertical axis shows power range (–20 to –100 dB).

In fact, our follow-up analysis on the power spectrum of original speech and locally time-reversed speech across all frequency range suggested that there was a significant difference between the original speech and locally time-reversed speech under some conditions (**Figure 6**). For analysis, two consecutive reversed segments of locally time-reversed speech were taken out, where the boundary of reversed segments sits in the middle, and, presumably, the additional noise or clicks at the boundary can be observed. The two consecutive segments taken out were the third and fourth reversed segments of all locally time-reversed sentences, because the number of reversals at each sentence varied depending on the reversed segment length applied (i.e., 10, 30, 50, 70, 90, or 110 ms). As all sentences could have minimum four times reversals with the full length of reversal applied, the current study focused on the third and fourth segments for the analysis of locally time-reversed speech. As for the original sentence, the corresponding part of speech was taken out for analysis. The average power spectrum across all frequency range was computed for 18 original sentences, and 18 locally time-reversed sentences with the reversed segment length of 10, 30, 50, 70, 90, and 110 ms respectively (i.e., 108 sentences in total), and the paired *t*-test was performed for each pair of the original speech and locally time-reversed speech. The results suggested that locally time-reversed speech and original speech was significantly different when the reversed segment length was 30 ms (*M* = -4.44, *SD* = 0.77, *t*(17) = -5.75, *p* < 0.001, 95% CI [-6.07 -2.81], *d* = 1.69), 50 ms (*M* = -3.72, *SD* = 0.87, *t*(17) = -4.29, *p* < 0.001, 95% CI [-5.54 -1.89], *d* = 2.06), and 70 ms (*M* = -5.35, *SD* = 1.07, *t*(17) = -5.00, *p* < 0.001, 95% CI [-7.60 -5.35], *d* = 2.88), while there was no significant difference when the reversed segment length was 10 ms (*M* = -1.12, *SD* = 0.57, *t*(17) = -1.95, *p* = 0.07, 95% CI [-2.33 0.09], *d* = 0.45), 90 ms (*M* = -0.65, *SD* = 1.36, *t*(17) = -0.47, *p* = 0.64, 95% CI [-3.52 2.23], *d* = 0.38), and 110 ms (*M* = -0.55, *SD* = 1.27, *t*(17) = -0.43, *p* = .67, 95% CI [-3.23 2.13], *d* = 0.31). The reason why the statistical difference between locally time-reversed speech and original speech was observed in some conditions (30, 50, and 70 ms) but not in other conditions (10, 90, and 110 ms) remains to be explored in the future study (along with the development of analysis methods). What the current study can say for now is that the additional artifacts, possibly generated at the boundary, can make the acoustic quality of locally time-reversed speech different from that of original speech.

On the other hand, the manipulation of local time reversal itself also creates the unnatural sequence of speech signal. That is, locally time-reversed speech itself is *not* following the natural articulatory dynamics at all. There are no natural transitions of formant frequencies not only at the edges of reversed segments but also inside every reversed segment. Everything is played backward in every reversed segment, which means, the possible motor actions in real life are not realized or represented in reversed speech. There is no segmental or suprasegmental information at all (e.g., phonemes, syllable, rhythms) in the natural order. If the consonant of “stop” is flipped, for example, the VOT is also flipped, and there is no natural articulatory movement represented in the speech signal. Also, there is no natural amplitude attenuation of speech (which takes place in the natural articulation from the opening of the mouth to the closing of the mouth) since every certain length of speech signal is flipped in time. In fact, locally time-reversed speech contains a lot of unnatural “acoustic” as well as “articulatory” artifacts that cannot be found in natural speech. The modulation frequency components of locally time-reversed speech are different from those in natural speech due to these additional artifacts generated by the local time reversal. Therefore, the regular occurrence of local time reversal, measured in time, cannot be simply discussed in the modulation frequency domain, just by directly converting the reversed segment length (e.g., 125 – 333 ms) into frequency (e.g., 3 – 8 Hz).

While two experiments of the current study exhibited the similar level of intelligibility as a function of reversed segment length (Experiment 1), and low-pass cut-off frequency (Experiment 2), there was also *no* direct correspondence between the reversed segment length (ms) and cut-off frequency (Hz) (i.e., preserved modulation frequency components) that regulated the temporal envelope of speech. To recall, native speakers of English understood speech with 97, 95, 64, 44, 24, and 23% accuracy when every 10, 30, 50, 70, 90, and 110 ms of speech was flipped in time, while the same individuals understood speech with 96, 95, 85, 44, 21, and 17 % accuracy when speech was low-pass filtered at a cut-off frequency of 32, 16, 8, 4, 2, and 1 Hz. In addition, non-native speakers of English understood speech with 51, 40, 20, 15, 9, and 10% accuracy when every 10, 30, 50, 70, 90, and 110 ms of speech was flipped in time, while the same individuals understood speech with 50, 44, 32, 19, 8, and 5% accuracy when speech was low-pass filtered at a cut-off frequency of 32, 16, 8, 4, 2, and 1 Hz. The similar level of intelligibility was observed across six levels of degradation in two different tasks, for native and non-native speakers respectively. However, there is no direct correspondence between the reversed segment length and low-pass cut-off frequency (i.e., preserved modulation frequency components). The reversed segment length of 10, 30, 50, 70, 90, and 110 ms corresponds to 100, 33, 20, 14, 11, and 9 Hz by simple conversion, and this does not match the modulation cut-off frequencies of 32, 16, 8, 4, 2, and 1 Hz. The modulation frequency that is involved in speech intelligibility cannot be simply computed by just looking at the critical reversed segment length of locally time-reversed speech, and converting the duration (ms) into frequency (Hz).

The lack of direct correspondence between the reversed segment length (ms) and modulation frequency (Hz) can be also possibly explained by the causality of acoustic degradation. As was discussed earlier, local time reversal drastically disrupts the temporal sequence of speech, and creates a new speech signal that contains additional acoustic and articulatory artifacts that do not exist in natural speech. On the other hand, modulation-filtered speech retains the natural acoustic and articulatory sequence of speech, at least, in the temporal order, although higher frequency components were eliminated when the low-pass cut-off frequency was applied. There are no additional noise or clicks in modulation-filtered speech, and there are no unnatural articulatory transitions in time as compared to the original speech except the fact that speech signal was low-pass filtered. In fact, local time reversal and modulation filtering are different types of acoustic degradation in its causality (i.e., whether the acoustic components are added to or reduced from the original speech signal), and, therefore, the critical modulation frequency components cannot be simply inferred from the intelligibility of locally time-reversed speech by converting the reversed segment duration (ms) into frequency (Hz).

On the other hand, the regular occurrence of local time reversal, measured in time, is also discussed in relation to linguistic properties in previous studies, as in whether speech perception takes place at the pace of syllables, phonemes, or both; or whether speech is perceived based on the accumulation of relatively short or long temporal information that is comparable to phoneme or syllable duration. Is this appropriate? While there is no one-to-one correspondence between the physical representation of speech signal and the mental representation of language, speech signal contains a lot of information to be decoded into different linguistic entities such as phonemes, syllables, words, phrases, rhythms, or intonation in a particular language. In fact, the stream of auditory input, which is physically represented as a continuous acoustic waveform, is linguistically understood as a sequence of words in the human brain. It is possible that a particular temporal property of speech signal, which is comparable to a particular articulatory gesture (and subsequently a particular linguistic entity), consists of the basic unit(s) of information to be integrated for speech perception (Liberman and Mattingly, 1985). However, it should be also kept in mind that the linguistic entities such as phonemes or syllables are what we perceive as a result of auditory processing, and these linguistic properties are no tangible entities at all in acoustic signals. In fact, there are no definite boundaries of a phoneme or a syllable in the physical surface of acoustic signal. Speech is always continuous, sustained by natural motor movements. In other words, no particular length of speech signal can be understood as a definite reflection of a particular linguistic entity such as a phoneme or a syllable with a definite boundary. The articulatory movement is continuous, especially in connected speech (Brown and Hilferty, 1982, 1986, 1995; Dalby, 1986; Johnson, 2004), and a phoneme or a syllable cannot be easily cut out just by defining them by an average duration of articulation. The acoustic cues for perceptual restoration is dispersed in a broad temporal range, and this helps perceptual restoration of speech as was also claimed in previous studies (Kashino et al., 1992; Kashino, 2006).

The conventional view of speech perception claims that the short-term spectra of speech, which linguistically correspond to consonants and vowels, are possibly the basic units of information to be integrated for speech perception, and these units are accumulated to make sense of speech (Liberman et al., 1967; Marslen-Wilson and Tyler, 1980; McClelland and Elman, 1986). On the other hand, recent research claims that speech perception does not require the detailed analysis of short-term spectra, but rather suprasegmental information such as syllables or rhythms (Huggins, 1975; Arai and Greenberg, 1997, 1998; Greenberg et al., 1998; Greenberg and Arai, 2001; Poeppel, 2003; Hickok and Poeppel, 2007; Stilp et al., 2010). Grataloup et al. (2009) examined the intelligibility of two-syllable French words, by locally time-reversing the first half syllable (0.5), one syllable (1), one-and-a-half syllables (1.5), or two syllables (2 = a word), and reported that the intelligibility of two-syllable French words drastically dropped when the first 1.5 syllables were locally time-reversed – a lack of syllabic information resulted in the lack of intelligibility. By this point of view and experimental procedure, it might be possible to explain a perceptual unit (e.g., syllable or phoneme) for speech perception in different languages (e.g., mora-, syllable-, or stress-timed languages as in Japanese, French, or English), but, again, it should be kept in mind that no linguistic unit can be cut out with the definite acoustic boundary as articulatory movement is always continuous. The relationship between physical acoustic properties, and perceptual linguistic entities remains largely to be explored. Additionally, Poeppel (2003) suggested, from the neuroscience point of view, that a cortical oscillation pattern which roughly matches the rate of syllable production seems to be critical for speech intelligibility. Further, the cortical oscillation which roughly matches the rate of phoneme production is also seemingly involved in speech perception. Again, a series of previous studies discusses the physical entities of speech signal or neural oscillation in relation to linguistic entities that people perceive, but there are no direct correspondence between the physical entities of speech signal (that can be measured in time with a specific boundary) and perceptual entities of linguistic units (that cannot be simply measured in time with a specific boundary).

Lastly, as limitations, this study examined the perceptual restoration of degraded speech by native and non-native speakers, and the language proficiency of non-native speakers was solely defined by the vocabulary size that was tested in the DIALANG Test. In the future study, language proficiency should be more carefully defined based on multiple dimensions, i.e., not only vocabulary size but also listening, reading, speaking, and writing proficiency. The measurement of L2 proficiency itself should be also further studied and developed in order to examine the relationship between language proficiency and perceptual restoration systematically. In addition, the current study collected data in different countries, and used the sound-proof rooms as well as available apparatus that are equivalent in function. The use of identical apparatus would be ideal in future studies, although, in reality as in the current study, the best effort that researchers can make is to use available resources by setting the conditions as equivalent as possible. The effective control of language proficiency as well as the use of identical apparatus in different countries should be kept in mind in future studies.

What the current study can support, with the current study design with the focus on the physical properties of speech signal, is that a lower modulation frequency (around 4 Hz) is likely to be critical for speech intelligibility, and the intelligibility of locally time-reversed speech drops when the reversed segment length is shorter than 100 ms (around 70 ms). Also, the perceptual restoration of temporally degraded speech was largely sustained by language proficiency (i.e., vocabulary size in the current study design). The relationship between the physical properties of speech signal (i.e., temporal duration, modulation frequency), and the perceptual entities (i.e., linguistic units) should be explored further in the future study, along with the involvement of language proficiency in the process of perceptual restoration.

## Conclusion

The current study imposed two different kinds of temporal distortion onto speech signal, in order to examine how native and non-native speakers perceptually restore temporally distorted speech. The speech signal was either locally time-reversed at every fixed duration (Experiment 1), or low-pass filtered at a particular cut-off frequency (Experiment 2). That is, the temporal envelope of speech was degraded as a function of reversed segment length, and as a function of low-pass cut-off frequency. The results suggest that speech becomes gradually unintelligible when every longer segment of speech is flipped in time, and when a lower cut-off frequency is imposed (i.e., when fewer modulation frequency components are preserved). Both native and non-native speakers exhibited the equivalent level of speech intelligibility in two different experiments across six levels of temporal distortion. With the current study design, the intelligibility of speech dropped by half when every 70 ms of speech was flipped in time (44% intelligibility), and when speech was low-pass filtered at a cut of frequency of 4 Hz (44% intelligibility). There was no direct correspondence between the reversed segment length, and low-pass cut-off frequency (i.e., preserved modulation frequency components), although the regular occurrence of local time reversal is often discussed in the modulation frequency domain. For now, the current study can conclude that the modulation frequency that is involved in speech intelligibility, cannot be computed or inferred by directly converting the length of critical reversed segment (ms) that is involved in the intelligibility of speech, into frequency (Hz).

## Ethics Statement

This study was carried out in accordance with the recommendations of the guidelines of the Institutional Review Board (IRB) of Stony Brook University in the United States and of NTT Communication Science Laboratories in Japan. The protocol was approved by the Institutional Review Board (IRB) of Stony Brook University and NTT Communication Science Laboratories. All subjects gave written informed consent in accordance with the Declaration of Helsinki.

## Author Contributions

MI, TA, and MK jointly designed the study. MI and TA constructed the stimuli. MI conducted the experiments, analyzed the data, and wrote a manuscript. TA and MK gave feedback to the manuscript for revision. All the authors read and approved the final manuscript.

## Conflict of Interest Statement

The authors declare that the research was conducted in the absence of any commercial or financial relationships that could be construed as a potential conflict of interest. The reviewer SC and handling Editor declared their shared affiliation.

## Figures and Tables

**Table A:** Sentences for Experiment 1.

Come and see me.
A blue one is little.
Where is it?
My yellow one is big.
And away we go.
You said to make it funny.
The red one is for you.
Now that was funny!
Please say yes.
Can you come out and play?
This is my new one.
I am good at this.
Do you like to run and jump?
It is a little too big for you.
I will look into it.
He is up to no good.
He will not get away with it.
I like the blue one on you.


**Table B:** Sentences for Experiment 2.

We can jump.
What did you find under there?
I can find it.
It is three to two.
They came to see our new one.
Look up here.
It is not in here.
We all ran to see what was there.
Will you help me down?
Help me down.
She said that they will be here soon.
Did you two eat?
It is so pretty up here!
Do you have this in white?
Red, yellow, and black make brown.
I must say, you ate well.
One and three are four.
Say what you want, but I like it.

