# Supplementary material for: Perceptual Restoration of Temporally Distorted Speech in L1 vs. L2: Local Time Reversal and Modulation Filtering
Source: Front Psychol. 2018 Sep 19;9:1749. doi: 10.3389/fpsyg.2018.01749 (PMC6156149; doi:10.3389/fpsyg.2018.01749)
Supplement: Supplementary file 2 [file Table_2.DOCX]

Table 2.

The summary of independent *t*-test for Experiment 2 (modulation-filtered speech).
